# The role of ClpP, RpoS and CsrA in growth and filament formation of *Salmonella enterica* serovar Typhimurium at low temperature

**DOI:** 10.1186/s12866-014-0208-4

**Published:** 2014-08-14

**Authors:** Gitte Maegaard Knudsen, Maj-Britt Nielsen, Line Elnif Thomsen, Søren Aabo, Ivan Rychlik, John Elmerdahl Olsen

**Affiliations:** 1 Department of Veterinary Disease Biology, Faculty of Health and Medical Sciences, University of Copenhagen, Stigbøjlen 4, Frederiksberg C 1870, Denmark; 2 National Food Institute, Technical University of Denmark, Mørkhøj Bygade 19, Søborg 2860, Denmark; 3 Veterinary Research Institute, Hudcova 70, Brno, 621 00, Czech Republic; 4 Present address: GMK: Department of Systems Biology, Technical University of Denmark, Matematiktorvet bldg 301, Kgs, Lyngby, 2800, Denmark; 5 Present address: MBN: DANSTEM, Faculty of Health and Medical Sciences, University of Copenhagen, Blegdamsvej 3B, Copenhagen N 2200, Denmark

**Keywords:** Salmonella, Cold adaptation, ClpP, RpoS, CsrA

## Abstract

**Background:**

*Salmonellae* are food-borne pathogens of great health and economic importance. To pose a threat to humans, *Salmonellae* normally have to cope with a series of stressful conditions in the food chain, including low temperature. In the current study, we evaluated the importance of the Clp proteolytic complex and the carbon starvation protein, CsrA, for the ability of *Salmonella* Typhimurium to grow at low temperature.

**Results:**

A *clpP* mutant was severely affected in growth and formed pin point colonies at 10°C. Contrary to this, *rpoS* and *clpP/rpoS* mutants were only slightly affected. The *clpP* mutant formed cold resistant suppressor mutants at a frequency of 2.5 × 10^−3^ and these were found not to express RpoS. Together these results indicated that the impaired growth of the *clpP* mutant was caused by high level of RpoS. Evaluation by microscopy of the *clpP* mutant revealed that it formed filamentous cells when grown at 10°C, and this phenotype too, disappered when *rpoS* was mutated in parallel indicating a RpoS-dependency. A *csrA* (sup) mutant was also growth attenuated a low temperature. An *rpoS/csrA* (sup) double mutant was also growth attenuated, indicating that the phenotype of the *csrA* mutant was independent from RpoS.

**Conclusions:**

The cold sensitivity of *clpP* mutant was associated with increased levels of RpoS and probably caused by toxic levels of RpoS. Although a *csrA* mutant also accumulated high level of RpoS, growth impairment caused by lack of *csrA* was not related to RpoS levels in a similar way.

## Background

Low temperature is one of the most extensively used methods to inhibit growth of pathogens and spoilage microorganisms, either in the form of rapid chilling or as long-term storages at cooling temperatures. The low temperatures cause decreases membrane fluidity and stabilizes secondary structures of RNA and DNA in the bacteria, which compromises membrane functions and cause a reduced efficiency in DNA replication, transcription and translation (Reviewed by Phadtare [1], Wouters *et al.*, [2]; Ramos *et al.*, [3]; Gualerzi *et al.*, [4] and Phadtare *et al.*[5]).

A number of stressful conditions can cause damage to and misfolding of proteins, and this has been shown to pose a threat to the bacterium. Degradation of abnormal proteins is dependent on proteases such as Lon and the Clp proteolytic complex [6]. The latter consists of the ClpP protease subunits where degradation takes place coupled with ClpX or ClpA ATPase/chaperone subunits responsible for substrate recognition, unfolding of proteins and translocation into the ClpP protease (reviewed by Gottesman [7]). Although misfolding of proteins is not a prominent feature of stress caused by temperature down shift [1], *Staphylococcus aureus* carrying mutations in the *clpP* and *clpX* genes are severely affected in formation of colonies at 17°C [8]. *clpP* is likewise essential for acclimation to growth below optimal temperature in other bacteria such as *Streptococcus pneumoniae*[9] and the cyanobacteria *Synechococcus*[10]. In *Bacillus thuringiensis,* the cell morphology is affected as *clpP1* mutants form filamentous cells at low temperatures indicating that ClpP1 is essential for cell separation [11]. In Gram negative bacteria, ClpP has been shown to be essential for virulence in both *Helicobacter pylori* and *Salmonella enterica*[12],[13], and deletion cause excess flagella production in *Salmonella*[14]. The amount of ClpP protein increases in *E. coli* during growth at 6 or 8°C, when compared to 15°C [15], which could imply a role in adaptation to cold environments, however, in general the role of this protease during adaptation to low temperature in Gram-negative bacteria remains unknown.

*Salmonella* is an important Gram-negative pathogen that causes gastroenteritis in humans and has major economic importance due to medical costs, lost productivity and recall of produce [16]. Human infections are predominantly caused by contaminated food and to pose a threat to humans, *Salmonella* has to pass and survive in the cooling processes of the food chain [17]. Based on the role of ClpP in cold shock adaptation in Gram-positive bacteria, this study hypothesized that ClpP is essential for growth and survival of *S. enterica* serovar Typhimurium (*S.* Typhimurium) at low temperatures. As the ClpP proteolytic complex, among other important functions, regulates the level of the stationary sigma factor, RpoS [18], an investigation of the importance of *rpoS* was performed in parallel. This enabled us to distinguish between the proteolytic effect of ClpP on misfolded proteins, and how this affected growth at low temperature, and the indirect effect of ClpP caused through degradation of RpoS.

Similar to the *clpP* mutant, we have previously shown that a mutant in the carbon starvation regulator protein gene, *csrA,* cause accumulation of high levels of RpoS [13]. Since we demonstrate in the current study that high level of RpoS in a *clpP* mutant appears to affect growth at low temperature, we hypothesised that a *csrA* mutant in a similar way would be growth attenuated, and included an investigation of this gene as well.

## Result and discussion

### A *clpP* mutant is impaired for growth at low temperature

**Figure 1:**
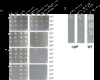
**ClpP and CsrA are important for growth at low temperature. A)***S.* Typhimurium C5 and isogenic mutants were grown exponentially in LB at 37°C up to an OD_600_ of 0.4. The cultures were then serially diluted (10^−1^-, 10^−2^-, 10^−3^-, and 10^−4^-fold), and 10 μl of each dilution was spotted onto LB plates. The plates were incubated at 10, 15, 21 and 37°C. The result presented is representative at least two experiments. **B)** The *clpP* are diluted as in a) and grown first at 10°C for 12 days and then transferred to 37°C for 1 day. A culture grown at 37°C for 1 day is included as control.

Growth of the *clpP* mutant was impaired on LB agar at 10°C (Figure 1A), whereas colony formation was delayed but resulted in normal size colonies at 15 and 21°C (Figure 1A). The temperature of 10°C was selected to represent the lower part of the temperature growth range of *S.* Typhimurium and still allow growth experiments to be carried out within a reasonable time. With increasing incubation time at 10°C, two growth phenotypes of the *clpP* mutant appeared: normal sized colonies and pin-point colonies. To test if the pin-point colonies were just small due to longer doubling time, the plate with the *clpP* mutant was transferred to 37°C after 12 days at 10°C, grown overnight and compared with wild type strain that had also grown overnight. Normal sized colonies were formed and the cell density corresponded to the wild type strain (Figure 1B). This showed that the *clpP* mutant was able to restore normal growth even after a long period at 10°C.

**Figure 2:**
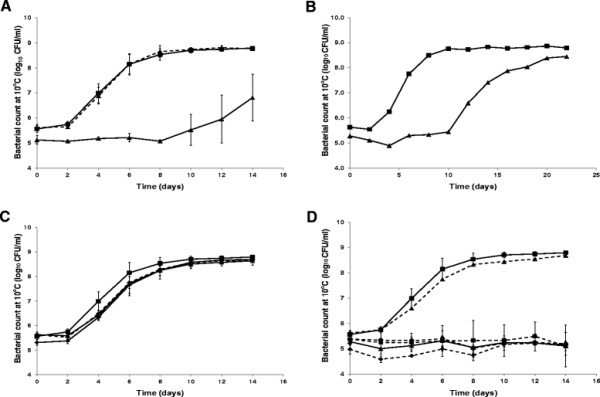
**Effect of ClpP, RpoS and CsrA on growth in LB at 10°C.** Overnight cultures were diluted 1000-fold in LB and incubated at 10°C without aeration. Growth was measured by enumeration on LB agar at 37°C. **A)** Growth of C5 (■, full line), *clpP* mutant (▲, full line) and *clpP*^*+*^ mutant (▲, broken line). **B)** Growth of C5 (■, full line), *clpP* mutant (▲, full line) for extend period. One biological replicate are shown. **C)** Growth of C5 (■, full line), *rpoS* mutant (▲, full line), *clpP/rpoS* mutant (♦, full line) and *clpP*^*+*^*/rpoS* mutant (♦, broken line). **D)** Growth of C5 (■, full line), *csrA* sup mutant (▲, full line), *csrA*^+^ sup mutant (▲, broken line), *clpP/csrA sup* (■, broken line), *rpoS/csrA* sup (●, broken line) and *clpP/rpoS/csrA* sup mutant (♦, broken line). The results are average of three independent biological replicates and SD are shown except *rpoS/csrA* sup and *clpP/rpoS/csrA* sup that were performed twice and *csrA*^+^ sup that were performed once.

The lag phase of the wild type C5 strain was 2.04 ± 0.66 days when grown in LB broth at 10°C, whereas the *clpP* mutant had a significantly longer lag phase of 9.97 ± 1.94 days (*p* = 0.002) (Figure 2A). The growth rate of the *clpP* mutant in exponential phase was 0.45 ± 0.03 days, which was a 29% reduction compared to the wildtype. The maximal density of the *clpP* mutant (8.29 log_10_ CFU/ml) was comparable to that of the wild type (8.74 log_10_ CFU/ml) after prolonged incubation (Figure 2B). To sustain that these phenotypes were not caused by secondary mutations, a wild-type *clpP* allele was re-introduced into the *clpP* mutant. Normal growth was restored by this complementation as neither growth rate nor lag phase were significantly altered compared to the wild type (*p* = 0.66 and *p* = 0.74; Figure 2A).

Normal size colonies of the *clpP* mutants were observed at 10°C with a frequency of 2.5 × 10^−3^ calculated as the difference in CFU count between normal sized colonies at 37°C and 10°C. By PCR, these were confirmed to contain the 240 bp deletion in the *clpP* gene and repeated growth at 10°C on LB agar plated confirmed a wild-type cold phenotype (data not shown). Based on the stability of the phenotype at 10°C and the presence of the deletion in the *clpP* gene, the colonies were assumed to be cold-resistant *clpP* suppressor-mutants. After growth at 10°C in liquid culture followed by spread on LB-agar at 37°C, 12 colonies were randomly selected, confirmed for the presence of the *clpP* mutation by PCR and regrown at 10°C on LB agar plates. They all had normal wild-type growth pattern indicating that cold-resistant suppressor mutants ended up dominating the planktonic culture at 10°C (data not shown).

### Impaired growth of the *clpP* mutant at low temperature is associated with high levels of RpoS

Levels of RpoS increase in *E. coli* at low temperature. This is due to an increase in the expression of the untranslated mRNA *dsrA,* which activates RpoS translation and cause induced expression of RpoS-dependent genes such as *bolA*[19]. Since RpoS is a substrate for the ClpXP proteolytic complex [18], mutation in *clpP* also leads to increased levels of RpoS [13]. Thus, we hypothesized that the increased RpoS levels caused by the cold temperature and the absence of RpoS degradation by ClpP proteolytic complex was responsible for the impaired growth of the *clpP* mutant. We therefore compared the growth of an *rpoS* and a double *clpP/rpoS* mutant to that of the *clpP* mutant. Both the *rpoS* mutant and the *clpP/rpoS* mutant grew at all temperatures tested and formed colonies similar to the wild type (Figure 1A). The lag phase of the *rpoS* and *clpP/rpoS* mutants were not significantly different from the wild type (*p* = 0.33 and 0.81) and growth rate did not differ, too (*p* = 0.74 and 0.0.94) (Figure 2C). This indicate that RpoS is not needed for growth of *S.* Typhimurium at low temperature and also that the growth attenuation at low temperature seen with the *clpP* mutant most likely was related to high levels of RpoS. Consistent with our observation, RpoS is not essential for growth at low temperature in *E. coli* in neither rich nor minimal medium [19]. The exact reason for the toxicity due to increased levels of RpoS in the *clpP* mutant remains elusive. A broad look at the effect, particularly on the RpoS regulon, can be obtained by use of global gene expression analysis, for example using DNA array, and such investigations are needed.

**Figure 3:**
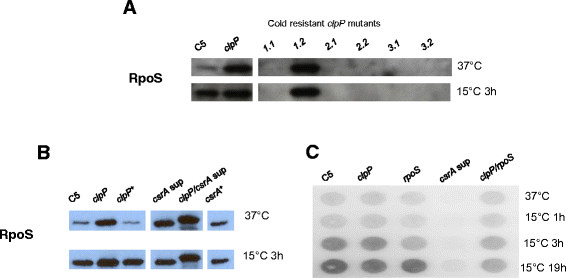
**The effect of the*clpP, rpoS*and*csrA*genes on the level of RpoS and expression of*csrA*.** Cells were grown to late log phase (OD_600_ of 0.65) in LB at 37°C or cold-shock at 15°C. **A)** The level of RpoS determined by Western blot in the wild type, *clpP* mutant and six cold resistant *clpP* suppressor mutants isolated from three independent experiments. Suppressor 1.1 and 1.2 was from the initial isolation of 12 random isolated. Suppressor 2.1 and 2.2 was from the quantification of suppressor frequency. Suppressor 3.1 and 3.2 was isolated at day 14 from other biological replicate of growth at 10°C. **B)** The level of RpoS determined by Western blot in the wild-type C5 and isogenic mutants before and after 3 hours of cold shock. **C)** The expression of *csrA* in the wild type and *clpP, rpoS, csrA* (sup) and *clpP/rpoS* mutants. RNA was extracted, dot blotted onto a hybridization filter and hybridized with labelled *csrA* probe. All figures are images of one representative gel where irrelevant samples have been edited away.

If our hypothesis that the high levels of RpoS were responsible for the growth defect in the *clpP* mutant at 10°C was correct, it was likely that the cold-resistant *clpP* suppressor mutants would have lower levels of RpoS than the *clpP* mutant. The cold-resistant *clpP* suppressor mutants from three independent experiments were tested by Western blot analysis for RpoS levels, and in five out of six strains with suppressor phenotype isolated from three different experiments, no RpoS was detected (Figure 3A). The sixth cold-resistant *clpP* suppressor mutant grew at low temperature and yet showed normal levels of RpoS. We do not currently have any explanation for this, and further studies are needed to investigate whether RpoS is actually functioning in this strain. As we saw the expected results in five out of six mutants, we considered this outside the scope of the current investigation. Genome sequencing of all the cold-resistant *clpP* suppressor-mutants would informative and are needed to identify which mutations that are the cause the suppressor mutants phenotype. Temperature down shift was shown to increase the RpoS level in the wild-type strain, and as expected, RpoS levels were higher in the *clpP* mutant than in the wild-type strain (Figure 3A and B).

RpoS levels at low temperature in *Salmonella* has not previously been investigated, however, the lack of a growth phenotype in the *rpoS* mutant in the current study corresponds well with previous results, showing that an *rpoS* mutant of *S.* Typhimurium SL1344 was only slightly sensitive to low temperature [20]. In contrast to results from *Listeria monocytogenes*, where *clpP* is expressed at elevated level when grown at 10°C [21], temperature down shift did not cause increased *clpP* transcription in *S.* Typhimurium (data not shown), and we interpret this as a further indication that the effect of ClpP deletion on growth a low temperature is indirect, i.e. caused by too high levels of RpoS.

### The *csrA* gene is essential for growth at low temperature independent of *clpP* and *rpoS*

The *csrA* gene was first identified in a screen of factors affecting glycogen accumulation [22], and a *csrA* mutant accumulates high amounts of glycogen [23]. More recently, it was found that glycogen accumulation is involved in protection against environmental stress similar to other sugar components [24]. The *csrA* system has been found to be important for numerous cell functions affecting virulence, motility and stress adaptation [25]–[27], and both deletion and over-expression of this gene have been shown to affect the cell morphology in *Legionella pneumophila* and *E. coli*[22],[28],[29]. Mutation of *csrA* causes severe growth defects at 37°C and suppressor mutants arise spontaneously [30],[31]. To overcome the uncertainty of working with a mixed population of original and spontaneous suppressor mutants, we have previously chosen to work with a Δ*csrA*::*kan* suppressor mutant [13], and the same well-characterized suppressor mutant was used in the present study.

The *csrA* (sup) mutant was severely impaired in colony formation on LB agar already at 21°C (Figure 1A) as well as during growth in LB broth at 10°C (Figure 2D). This phenotype could be reversed by complementation of the *csrA* gene (Figure 2D) and further by using an arabinose inducible promoter (Additional file 1: Figure S1). Unlike the *clpP/rpoS* double mutant*,* the *rpoS/csrA* (sup) mutant did not grow at 21°C nor at lower temperatures (Figure 1A), indicating that the *csrA* gene was essential for growth at low temperature independent from RpoS levels. Growth of the *clpP/csrA* mutant was similarly impaired, however, the ability of this strain to grow a low temperature increased slightly compared to the *csrA* (sub) mutant (growth possible at 21°C and a 15°C). This improvement disappeared when *rpoS* was mutated in addition to *clpP* and *csrA* (Figures 1 and 2). As both the mutation in *clpP* and *csrA* cause increased RpoS level, one could have expected growth to be more affected. We investigated if the level of RpoS was increased in the double mutant. As previously reported [13], the RpoS level was increased both in the *clpP* and *csrA* mutants at 37°C, and further it increased when transferred to 15°C for 3 h (Figure 3B). The RpoS protein detected in the *clpP/csrA* mutant, however, was clearly larger when compared to the protein of the wild type and single mutants, indicating changes in the protein. We propose that RpoS does not function correctly in this strain, and that this allow the strain to cope with the mutations. Since we observed an elevated level of RpoS protein with apparent normal size in the *csrA* (sup) mutant, the negative growth effect of RpoS is likely to be present in this strain too. However, the growth defect caused by lack of CsrA appears to be stronger since the double mutant remains severely growth affected.

### Expression of *csrA* is increased during growth at 15°C

To get further insight into the essential role of *csrA* at low temperature, we investigated whether this gene was expressed at elevated levels at low temperatures. Expression of *clpP* was included as a control, and the expression of this gene was not altered after a temperature downshift to 15°C compared to 37°C (data not shown). In contrast, the expression of *csrA* was increased several fold in the wild type and *clpP* mutants, both at 3 and 19 hours after the temperature downshift (Figure 3C), This supports that CsrA plays a specific role in adaptation to growth at low temperature. In the *rpoS* mutant after 3 hours, and in the *clpP/rpoS* double mutant after both 3 and 19 hours, expression of *csrA* was lower than in the other strains tested. After 3 hours, the level in the double mutant corresponded to the level in the *rpoS* mutant. *csrA* expression is controlled by RpoS at 37°C [13], and the results are consistent with this also being the case at 10°C. Why the control appears to be lost after 19 hours in the single mutant is currently unknown, but it suggest that another mechanism steps in at this time point.

CsrA has previously been shown to be important for induction of the typical heat shock response in *Helicobacter pylori*[32]. Combined with our results, this could indicate that the CsrA protein is involved in temperature-dependent regulation both at high and low temperature, however, this has to be further investigated.

### *clpP*-mutation causes formation of filamentous cells in an RpoS dependent manner

Growth by elongation of cells with incomplete separation is important in relation to food safety. Rapid completion of separation occur when filamentous cells, produced during chilling, are transferred to 37°C, and a more than 200-fold increase in cell number can be found within four hours [33]. *S.* Enteritidis wild-type strains with normal RpoS level have previously been reported to produce filaments up to 150 μm at 10°C whereas strains with impaired RpoS expression are only up to 35 μm long [33],[34].

**Figure 4:**
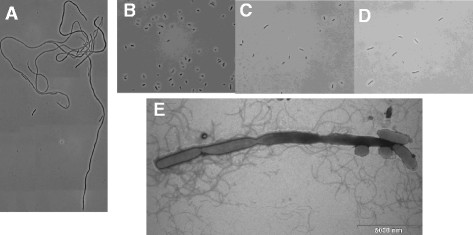
**The*clpP*mutant forms filaments during growth at 10°C.** Overnight cultures of *S.* Typhimurium C5 and mutants were diluted 1000-fold in LB and incubated at 10°C for 12 days without aeration and phase contrast microscopy pictures at 1000X manification were produced. **A)***clpP*, **B)** wild type, **C)***clpP*^*+*^, **D)***clpP/rpoS*. **E)** Electron microscopy picture of the *clpP* mutant after growth at 12°C for 14 days.

Microscopic examination of cultures grown at 10°C and 15°C showed that the *clpP* mutant formed long filamentous cells (Figure 4A) similar to what is seen for the *B. thuringiensis clpP1* mutant at 25°C [11]. In contrast, the wild type (Figure 4B) and the *clpP* mutant complemented with wild-type *clpP* allele (Figure 4C) formed cells of normal size; however, slightly longer at day 12 compared to a 37°C overnight cultures. The *clpP/rpoS* mutant lacked filament formation (Figure 4D).

By following the development of the *clpP* mutant during the growth experiment at 10°C, it was found that the length of the filaments formed by the *clpP* mutant increased over time and by day 10 only filamentous cells were observed. After this time point, the cell size became more heterogeneous in the population (data not shown). Electron microscopy of the *clpP* mutant revealed that at this stage the filaments were like cocktail sausages on a string (Figure 4E) indicating that septum formation had started but could not be completed.

The fact that only the *clpP* mutant of *S.* Typhimurium with high levels of RpoS formed filament at 10°C and 15°C, whereas the wild-type and the *clpP/rpoS* mutated strains showed normal cell size, indicates that filament formation is associated high levels of RpoS in *S.* Typhimurium. A possible explanation relates to the level of the cell division protein FtsZ, which is reported to be controlled by RpoS in *E. coli*[35], and to be a substrate for the ClpXP proteolytic complex [36],[37]. Further studies such as transcriptomic or proteomic analysis comparing the expression/protein profile of FtsZ in the wild type to expression in *clpP*, *clpP/rpoS* and *csrA* mutants are needed to further investigate the cold response.

## Conclusions

The findings presented in this report demonstrate new phenotypes related to the ClpP protease and the CsrA protein during growth at low temperatures. Although mutants in both genes accumulate high levels of RpoS, the mechanisms for lack of growth seem to be different. The results indicate that CsrA is essential for adaptation to growth at low temperature, in its own right, whereas the impaired growth of the *clpP* mutant is associated with the effect of elevated RpoS levels.

## Methods

### Bacterial strains and growth conditions

The bacterial strains used in this study are listed in Table 1. Overnight cultures were grown aerobically in LB broth, Lennox (Oxoid) at 37°C with agitation and stored in LB broth containing 15% glycerol at −80°C. To prepare cultures, frozen stock cultures were inoculated on LB agar and grown at 37°C overnight. Antibiotics (Sigma) were used when appropriate in the following concentrations: 50 μg ml^−1^ ampicillin, 50 μg ml^−1^ kanamycin, 20 μg ml^−1^ streptomycin and 100 μg ml^−1^ spectomycin.

**Table 1:** Bacterial strains and plasmids used in the study

**Strain or plasmids**		**Genotype and relevant characteristics**	**Reference**
*Salmonella* strains			
C5		*S.* Typhimurium*,* virulent wild type	[38]
*clpP*	LT1100	C5 Δ*clpP*	[39]
*clpP*^*+*^	LT1102	LT1100 with Tn*10* linked to *clpP*^+^ (linkage 48%)	[39]
*clpP/rpoS*	LT1104	LT1100 *rpoS*::Ap	[39]
*rpoS*	LT1105	C5 *rpoS*::Ap	[39]
*clpP*^*+*^*/rpoS*	LT1108	LT1102 *rpoS*::Ap	[39]
*csrA* (sup)	GMK201	C5 *csrA*::Kn sup, suppressor of *csrA* growth defect	[13]
*rpoS/csrA* (sup)	GMK206	LT1105 *csrA*::Kn, sup, suppressor of *csrA* growth defect	[13]
*clpP/rpoS/csrA* (sup)	GMK207	LT1104 *csrA*::Kn, sup, suppressor of *csrA* growth defect	[13]
*csrA*^+^ (sup)	GMK209	GMK201 with plasmid pCA132	[13]
Plasmids			
pCA132		0.7-kb *csrA* fragment on pFF584; Str^r^ Sp^r^	[30]

To investigate growth in broth, overnight cultures were diluted 5000-fold and incubated at 37°C with agitation. Growth was measured by optical density at 600 nm (OD_600_). To investigate growth on solid agar at low temperature, cells were grown until OD_600_ 0.4. Ten μl of a 10-fold dilution of the cultures were spotted on LB agar and incubated at different temperatures: 10, 15, 21, 25, 30, 37 and 42°C. Growth in LB broth at 10°C was investigated by making a 10-fold dilution of overnight culture. 40 μl of the 10^−1^ dilutions were inoculated in 40 ml LB broth. The culture were incubated at 10°C and at different time points, growth was measured by optical density and CFU enumeration by spotting of 10 μl of 10-fold serial dilutions on LB agar. To estimate the number of *clpP* cold suppressor mutants, serial dilutions of mutant and wild-type bacteria were plated on LB agar and incubated in parallel at 10 and 37°C. The growth parameters were estimated by the Baranyi growth equation [40] using the Excel macro DMFit (http://www.ifr.ac.uk/safety/dmfit). The average and standard deviation between the biological replicates were determined in Microsoft Excel.

### Microscopic investigation

Bacterial morphology was studied by phase contrast microscopy and by electron microscopy. Bacterial cultures for microscopy were grown as described above at low temperature. A drop of cultures were applied directly to microscope slides and observed by phase-contrast microscopy with a Zeiss Axioplan2 Microscope. For electron microscopy, bacterial cultures were grown in LB broth at 12°C. A drop of LB broth was placed onto 800-mesh copper grid, and excess liquid was removed after 10 min by filter paper. The grid was stained with 1% aqueous phosphotungstic acid (pH 7.0) for 60 s. The grid was examined with a transmission electron microscope Philips EM2085. Both for phase contrast and electron microscopy concentration by centrifugation of the *clpP* mutant were necessary.

### Western blot analysis

For analysis of intracellular expression of RpoS in normally grown and cold-shocked cells, bacteria were first grown in LB broth with aeration to OD_600_ 0.65 at 37°C. Once the cultures reached OD_600_ 0.65, control samples were prepared by centrifugation of 2 ml cultures and the remaining culture were quickly cooled on ice and moved to 15°C in a water-bath with moderate shaking. Cold-shock samples were taken after 1, 3 and 19 hours of incubation at 15°C. Cells were stored at −80°C until analysis. Cell pellets were suspended in lysis buffer (50 mM Tris–HCl (pH 8.0), 100 mM NaCl, 5 mM DTT, 1 mM PMSF) and lysed by FastPrep FP120 instrument (BIO101, ThermoSavent) by 5 rounds of 30 second at speed 6.5 followed by 2 min on ice. Cell debris was removed by centrifugation at 8,000 rpm for 15 min.

The protein concentration was determined by using a Bio-Rad protein assay (Bio-Rad Laboratories), and 5 μg of each sample was separated on NuPAGE 4 to 12% Bis-Tris gels (Invitrogen) using MOPS buffer (Invitrogen). The gels were stained with Coomassie blue using Safestain (Invitrogen) to check for equal amounts of protein or transferred onto a polyvinylidene difluoride membrane (Invitrogen) using an XCell SureLock Mini-Cell system (Invitrogen) as recommended by the supplier. RpoS was detected using *E. coli* RpoS monoclonal antibodies (NeoClone Biotechonolgy) at a 1:1000 dilution and the WesternBreeze Chemiluminescent Anti-Mouse kit (Invitrogen).

### RNA purification and dot blotting

For transcriptional analysis, RNA was purified from exponential grown and cold-shocked cells as described for Western blot analysis. The cells were harvested by centrifugation at 10,000 × *g* for 2 min and the pellet was stored at −80°C. RNA purification was performed using RNeasy Mini kit as described by Thomsen *et al.*[41]. RNA was quantified by measuring absorbance at 260 nm and quality was verified by 260 nm/280 nm as well as RNA was run on a agarose gel. Five μg of total RNA was loaded on the gel, and controlled for equal amounts loaded by staining with ethidium bromide. Three μg of total RNA were denatured as described by Frees *et al.*[42] and used for Dot blotting using a Minifold (Schleicher & Schuell) as described by Sambrook *et al.*[43] with minor modifications. Hybridization probes were generated by PCR from chromosomal DNA of *S.* Typhimurium C5 using specific primers for the *clpP* (5’-atgtcatacagcggagaacg and 5’-agattgacccgtatgatgcgc)*, rpoS* (5’- aacgacctggctgaagaaga and 5’- tcgttgagacgaagcatacg) and *csrA* (5’- atgctgattctgactcgtcg and 5’- ttagtaactggactgctggg) genes. The probes were labelled with [α-^32^P]dCTP, and hybridization was visualized with a STORM 840 Phosphorimager (Molecular Dynamics).

### PCR for detection of the *clpP* and *rpoS* genes

PCR for detection of the *rpoS* gene including a 600 bp upstream and 30 bp down-stream region of the gene was performed by standard procedures [43] with the following primers RpoS_F2 (5’- attctgagggctcaggtgaa) and RpoS_R2 (5’-cagtcgacagactggccttt). PCR for detection of *clpP* was performed using the primers ClpP-B1 (5′-agtagatctcgtctgcttacgaagatcc-3′) and ClpX-H1 (5′-cctaagcttacgccattgctggtatcg-3′).

## Abbreviations

CFU: Colony forming units

## Competing interests

The authors declare that they have no competing interests.

## Authors’ contributions

GMK, LETH, SABO and JEO planned the experiments. GMK performed growth experiments and western blots, GMK and MBN performed expression studies, GMK and IVR performed microscopy studies; GMK, JEO drafted the manuscript and all authors read and commented on this. All authors approved the final manuscript.

## Additional file

## Supplementary Material

Additional file 1: Figure S1. Complementation of the *csrA* (sup) mutant for growth at 10°C by introduction of the *csrA* gene in *trans* (pCA132) and further by using an arabinose inducible promoter (pC114 arabionose). Click here for file

## References

[B1] Phadtare S Recent developments in bacterial cold-shock response Cur Issues Mol Biol 2004 6 125 136 15119823

[B2] Wouters JA Rombouts FM Kuipers OP de Vos WM Abee T The role of cold-shock proteins in low-temperature adaptation of food-related bacteria Syst Appl Microbiol 2000 23 165 173 10.1016/S0723-2020(00)80001-6 10930067

[B3] Ramos JL Gallegos M-T Marqués S Ramos-González M-I Espinosa-Urgel M Segura A Responses of Gram-negative bacteria to certain environmental stressors Curr Opin Microbiol 2001 4 166 171 10.1016/S1369-5274(00)00183-1 11282472

[B4] Gualerzi CO Giuliodori AM Pon CL Transcriptional and post-transcriptional control of cold-shock genes J Mol Biol 2003 331 527 539 10.1016/S0022-2836(03)00732-0 12899826

[B5] Phadtare S Alsina J Inouye M Cold-shock response and cold-shock proteins Curr Opin Microbiol 1999 2 175 180 10.1016/S1369-5274(99)80031-9 10322168

[B6] Wickner S Maurizi MR Gottesman S Posttranslational quality control: folding, refolding, and degrading proteins Science 1999 286 1888 1893 10.1126/science.286.5446.1888 10583944

[B7] Gottesman S Proteolysis in bacterial regulatory circuits Annu Rev Cell Dev Biol 2003 19 565 587 10.1146/annurev.cellbio.19.110701.153228 14570582

[B8] Frees D, Qazi SN, Hill PJ, Ingmer H: **Alternative roles of ClpX and ClpP in*****Staphylococcus aureus*****stress tolerance and virulence.** *Mol Microbiol* 2003, **48:**1565–1578. 10.1046/j.1365-2958.2003.03524.x 12791139

[B9] Robertson GT, Ng WL, Foley J, Gilmour R, Winkler ME: **Global transcriptional analysis of*****clpP*****mutations of type 2*****Streptococcus pneumoniae*****and their effects on physiology and virulence.** *J Bacteriol* 2002, **184:**3508–3520. 10.1128/JB.184.13.3508-3520.2002 PMC135132 12057945

[B10] Porankiewicz J, Schelin J, Clarke AK: **The ATP-dependent Clp protease is essential for acclimation to UV-B and low temperature in the cyanobacterium*****Synechococcus*****.** *Mol Microbiol* 1998, **29:**275–283. 10.1046/j.1365-2958.1998.00928.x 9701820

[B11] Fedhila S, Msadek T, Nel P, Lereclus D: **Distinct*****clpP*****genes control specific adaptive responses in*****Bacillus thuringiensis*****.** *J Bacteriol* 2002, **184:**5554–5562. 10.1128/JB.184.20.5554-5562.2002 PMC139615 12270812

[B12] Loughlin MF, Arandhara V, Okolie C, Aldsworth TG, Jensk PJ: ***Helicobacter pylori*****mutants defective in the clpP ATP-dependent protease and the chaperone clpA display reduced macrophage and murine survival.** *Microb Pathog* 2009, **46:**53–57. 10.1016/j.micpath.2008.10.004 18992803

[B13] Knudsen GM, Olsen JE, Aabo S, Barrow P, Rychlik I, Thomsen LE: **ClpP deletion causes attenuation of*****Salmonella*****Typhimurium through mis-regulation of RpoS and indirect control of CsrA and the SPI genes.** *Microbiology* 2013, **159:**1497–1509. 10.1099/mic.0.065797-0 23676436

[B14] Tomoyasu T, Ohkishi T, Ukyo Y, Tokumitsu A, Takya A, Suzuki M, Sekiya K, Matsui H, Kutsukake K, Yamamoto T: **The ClpXP ATP-dependent protease regulates flagella synthesis in*****Salmonella enterica*****serovar Typhimurium.** *J Bacteriol* 2002, **184:**645–653. 10.1128/JB.184.3.645-653.2002 PMC139528 11790733

[B15] Jones TH, Murray A, Johns M, Gill CO, McMullen ML: **Differential expression of proteins in cold-adapted log-phase cultures of*****Escherichia coli*****incubated at 8, 6 or 2 degrees C.** *Int J Food Microbiol* 2006, **107:**12–19. 10.1016/j.ijfoodmicro.2005.08.006 16256234

[B16] Roberts JA Cumberland P Sockett PN Wheeler J Rodrigues LC Sethi D Roderick PJ The study of infectious intestinal disease in England: socio-economic impact Epidemiol Infect 2003 130 1 11 10.1017/S0950268802007690 12613740 PMC2869933

[B17] Humphrey TJ: ***Salmonella*****, stress responses and food safety.** *Nat Rev Microbiol* 2004, **2:**504–509. 10.1038/nrmicro907 15152206

[B18] Webb C, Moreno M, Wilmes-Riesenberg M, Curtiss R III, Foster JW: **Effects of DksA and ClpP protease on sigma S production and virulence in*****Salmonella typhimurium*****.** *Mol Microbiol* 1999, **34:**112–123. 10.1046/j.1365-2958.1999.01581.x 10540290

[B19] Sledjeski DD, Gupta A, Gottesman S: **The small RNA, DsrA, is essential for the low temperature expression of RpoS during exponential growth in*****Escherichia coli*****.** *EMBO J* 1996, **15:**3993–4000. PMC452119 8670904

[B20] McMeechan A, Roberts M, Cogan TA, Jørgensen F, Stevenson A, Lewis C, Rowley G, Humphrey TJ: **Role of the alternative sigma factors RpoE and RpoS in survival of*****Salmonella enterica*****serovar Typhimurium during starvation, refrigeration and osmotic shock.** *Microbiology* 2007, **153:**263–269. 10.1099/mic.0.29235-0 17185555

[B21] Liu S, Graham JE, Bigelow L, Morse PD, Wilkinson BJ: **Identification of*****Listeria monocytogenes*****genes expressed in response to growth at low temperature.** *Appl Environ Microbiol* 2002, **68:**1697–1705. 10.1128/AEM.68.4.1697-1705.2002 PMC123842 11916687

[B22] Romeo T, Gong M, Liu MY, Brun-Zinkernagel AM: **Identification and molecular characterization of*****csrA*****, a pleiotropic gene from*****Escherichia coli*****that affects glycogen biosynthesis, gluconeogenesis, cell size, and surface properties.** *J Bacteriol* 1993, **175:**4744–4755. 10.1128/jb.175.15.4744-4755.1993 PMC204926 8393005

[B23] Yang H, Liu MY, Romeo T: **Coordinate genetic regulation of glycogen catabolism and biosynthesis in*****Escherichia coli*****via the CsrA gene product.** *J Bacteriol* 1996, **178:**1012–1017. 10.1128/jb.178.4.1012-1017.1996 PMC177760 8576033

[B24] McMeechan A, Lovell MA, Cogan TA, Marston KL, Humphrey TJ, Barrow PA: **Glycogen production by different*****Salmonella enterica*****serotypes: contribution of functional*****glgC*****to virulence, intestinal colonization and environmental survival.** *Microbiology* 2005, **151:**3969–3977. 10.1099/mic.0.28292-0 16339941

[B25] Romeo T Global regulation by the small RNA-binding protein CsrA and the non-coding RNA molecule CsrB Mol Microbiol 1998 29 1321 1330 10.1046/j.1365-2958.1998.01021.x 9781871

[B26] Wei B, Shin S, LaPorte D, Wolfe AJ, Romeo T: **Global regulatory mutations in*****csrA*****and*****rpoS*****cause severe central carbon stress in*****Escherichia coli*****in the presence of acetate.** *J Bacteriol* 2000, **182:**1632–1640. 10.1128/jb.182.6.1632-1640.2000 PMC94461 10692369

[B27] Fortune DR, Suyemoto M, Altier C: **Identification of CsrC and characterization of its role in epithelial cell invasion in*****Salmonella enterica*****serovar Typhimurium.** *Infect Immun* 2006, **74:**331–339. 10.1128/IAI.74.1.331-339.2006 PMC1346597 16368988

[B28] Fettes PS, Forsbach-Birk V, Lynch D, Marre R: **Overexpresssion of a*****Legionella pneumophila*****homologue of the*****E. coli*****regulator*****csrA*****affects cell size, flagellation, and pigmentation.** *Int J Med Microbiol* 2001, **291:**353–360. 10.1078/1438-4221-00141 11727819

[B29] Forsbach-Birk V, McNealy T, Shi C, Lynch D, Marre R: **Reduced expression of the global regulator protein CsrA in*****Legionella pneumophila*****affects virulence-associated regulators and growth in*****Acanthamoeba castellanii*****.** *Int J Med Microbiol* 2004, **294:**15–25. 10.1016/j.ijmm.2003.12.003 15293450

[B30] Altier C, Suyemoto M, Lawhon SD: **Regulation of*****Salmonella enterica*****serovar Typhimurium invasion genes by csrA.** *Infect Immun* 2000, **68:**6790–6797. 10.1128/iai.68.12.6790-6797.2000 PMC97782 11083797

[B31] Martinez LC, Yakhnin H, Camacho MI, Georgellis D, Babitzke P, Puente JL, Bustamante VH: **Integration of a complex regulatory cascade involving the SirA/BarA and Csr global regulatory systems that controls expression of the*****Salmonella*****SPI-1 and SPI-2 virulence regulons through HilD.** *Mol Microbiol* 2011, **80:**1637–1656. 10.1111/j.1365-2958.2011.07674.x PMC3116662 21518393

[B32] Barnard FM, Loughlin MF, Fainberg HP, Messenger MP, Ussery DW, Williams P, Jenks PJ: **Global regulation of virulence and the stress response by CsrA in the highly adapted human gastric pathogen*****Helicobacter pylori*****.** *Mol Microbiol* 2004, **51:**15–32. 10.1046/j.1365-2958.2003.03788.x 14651608

[B33] Mattick KL, Phillips LE, Jørgensen F, Lappin-Scott HM, Humphrey TJ: **Filament formation by*****Salmonella*****spp. inoculated into liquid food matrices at refrigeration temperatures, and growth patterns when warmed.** *J Food Prot* 2003, **66:**215–219. 10.4315/0362-028x-66.2.215 12597479

[B34] Phillips LE, Humphrey TJ, Lappin-Scott HM: **Chilling invokes different morphologies in two*****Salmonella enteritidis*****PT4 strains.** *J Appl Microbiol* 1998, **84:**820–826. 10.1046/j.1365-2672.1998.00417.x 9674136

[B35] Cam K, Cuzange A, Bouche JP: **Sigma S-dependent overexpression of*****ftsZ*****in an*****Escherichia coli*****K-12*****rpoB*****mutant that is resistant to the division inhibitors DicB and DicF RNA.** *Mol Gen Genet* 1995, **248:**190–194. 10.1007/BF02190800 7651342

[B36] Flynn JM Neher SB Kim YI Sauer RT Baker TA Proteomic discovery of cellular substrates of the ClpXP protease reveals five classes of ClpX-recognition signals Mol Cell 2003 11 671 683 10.1016/S1097-2765(03)00060-1 12667450

[B37] Weart RB Nakano S Lane BE Zuber P Levin PA The ClpX chaperone modulates assembly of the tubulin-like protein FtsZ Mol Microbiol 2005 57 238 249 10.1111/j.1365-2958.2005.04673.x 15948963 PMC5432201

[B38] Hormaeche CE: **Natural resistance to*****Salmonella typhimurium*****in different inbred mouse strains.** *Immunology* 1979, **37:**311–318. PMC1457519 381178

[B39] Thomsen LE, Olsen JE, Foster JW, Ingmer H: **ClpP is involved in the stress response and degradation of misfolded proteins in*****Salmonella enterica*****serovar Typhimurium.** *Microbiology* 2002, **148:**2727–2733. 10.1099/00221287-148-9-2727 12213919

[B40] Baranyi J Roberts TA A dynamic approach to predicting bacterial growth in food Int J Food Microbiol 1994 23 277 294 10.1016/0168-1605(94)90157-0 7873331

[B41] Thomsen LE, Gottlieb CT, Gottschalk S, Wodskou TT, Kristensen HH, Gram L, Ingmer H: **The heme sensing response regulator HssR in*****Staphylococcus aureus*****but not the homologous RR23 in*****Listeria monocytogenes*****modulates susceptibility to the antimicrobial peptide plectasin.** *BMC Microbiol* 2010, **10:**307. 10.1186/1471-2180-10-307 PMC3001719 21122114

[B42] Frees D, Sørensen K, Ingmer H: **Global virulence regulation in*****Staphylococcus aureus*****: pinpointing the roles of ClpP and ClpX in the*****sar/agr*****regulatory network.** *Infect Immun* 2005, **73:**8100–8108. 10.1128/IAI.73.12.8100-8108.2005 PMC1307069 16299304

[B43] Sambrook J Fritsch EF Maniatis T Molecular Cloning: A Laboratory Manual 1989 Cold Spring Harbor Laboratory Press, Cold Spring Harbor, New York

