# Supplementary figures and images for: The role of ClpP, RpoS and CsrA in growth and filament formation of Salmonella enterica serovar Typhimurium at low temperature
Source: BMC Microbiol. 2014 Aug 14;14:208. doi: 10.1186/s12866-014-0208-4 (PMC4236599; doi:10.1186/s12866-014-0208-4)

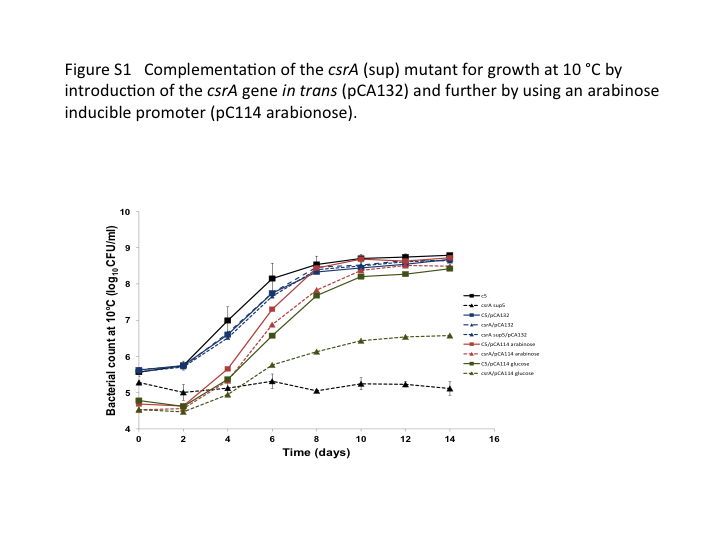

Supplement: Additional file 1: Figure S1. — Complementation of the csrA (sup) mutant for growth at 10°C by introduction of the csrA gene in trans (pCA132) and further by using an arabinose inducible promoter (pC114 arabionose). [file s12866-014-0208-4-S1.png]
